# Individual and household correlates of *Helicobacter pylori* infection among Young Ethiopian children in Ziway, Central Ethiopia

**DOI:** 10.1186/s12879-020-05043-1

**Published:** 2020-04-25

**Authors:** Kayla Schacher, Hannah Spotts, Caroline Correia, Sosina Walelign, Mehret Tesfaye, Kassu Desta, Aster Tsegaye, Bineyam Taye

**Affiliations:** 1 Department of Biology, Colgate University, 214 Olin Hall, 13 Oak Dr., Hamilton, NY 13346 USA; 2 Addis Ababa University, College of Health Sciences, Department of Medical Laboratory Science, Addis Ababa, Ethiopia

**Keywords:** *H. pylori*, Risk factors, Children, Ethiopia

## Abstract

**Background:**

Investigating distinct individual- and household-level risk factors for acquiring *Helicobacter pylori* (*H. pylori*) infection can inform disease prevention efforts and implicate possible routes of transmission. This study determined the magnitude of *H. pylori* infection among schoolchildren in Ziway, central Ethiopia and identified personal and household correlates of *H. pylori* infection in young Ethiopian children.

**Methods:**

A total of 434 schoolchildren participated in this cross-sectional study. Infection status was assessed using antigen and antibody rapid tests. Demographic and lifestyle information was obtained from parents via an interviewer-led questionnaire. Univariate and multivariate logistic regressions were performed to assess the relationships between potential individual- and household-level risk factors and *H. pylori* infection.

**Results:**

The prevalence of *H. pylori* infection was 65.7% (285/434). Of the personal variables assessed, the age group 10–14 years was found to be significantly associated with higher odds of *H. pylori* infection in univariate analysis (COR = 2.22, 95% CI: 1.06–4.66, *p* = 0.03) and remained positively correlated after adjusting for confounding factors. Of the household-level factors explored, having a traditional pit or no toilet was found to be significantly associated with 3.93-fold higher odds of *H. pylori* infection (AOR = 3.93, 95% CI: 1.51–10.3, *p* = 0.01), while the presence of smokers in the household was associated with 68% lower odds of infection (AOR = 0.32, 95% CI: 0.11–0.89, p = 0.03).

**Conclusion:**

This study from a developing country provides additional evidence for older age as a personal risk factor for *H. pylori* infection and identifies correlations between socioeconomic and sanitation household factors and positive childhood infection status. The associations reported here support the hypothesized fecal-oralroute of transmission for *H. pylori.*

## Background

*Helicobacter pylori* (*H. pylori*) is a pathogenic bacterium that infects human gastric mucosa [1]. It is known to be one of the leading causes of dyspepsia, active gastritis, peptic ulcers, and gastric adenocarcinomas [1]. Because of its role in gastric cancers, it is classified as a World Health Organization Group 1 carcinogen [2]. Nearly one half of the global population is infected with *H. pylori*, with some countries reporting over 80% infection rates [3, 4]. Although prevalence rates have been generally declining in recent years, infection remains more prevalent in developing countries in comparison to developed countries [3, 4]. The reasons for this are not yet fully established, though are speculated to be related to poorer sanitation and living conditions in resource-limited settings [3].

Close person-to-person contact, especially between family members living together, has been shown to be important for *H. pylori* transmission [5, 6]. The exact mode of person-to-person transmission remains unclear though, with literature proposing both oral-oral and fecal-oral routes. Although *H. pylori* has been detected in dental plaque, saliva, gastric juice, and fecal matter, samples outside of the stomach have proven difficult to culture [7, 8]. Identifying associated risk factors for infection can create a more comprehensive understanding of probable *H. pylori* transmission mechanisms.

Most *H. pylori* infections are acquired during childhood and persist into adulthood [9]. Infection often remains latent for years, with symptomatic disease developing up to several decades later [1]. Accordingly, *H. pylori* transmission is more highly associated with childhood living conditions than adult living conditions [9, 10]. Previous studies have thus sought to identify select risk factors for *H. pylori* infection by examining childhood environments [10–15]. Common risk factors examined in these studies include age, indicators of socioeconomic status, and poor hand and cooking sanitation [10–16]. Both individual and familial characteristics have been shown to influence infection status. Investigating separate personal- and household-level risks for acquiring *H. pylori* infection can elucidate appropriate levels of disease prevention efforts. While personal attributes are crucial to investigate, factors affecting entire households have a greater impact on the health of families and groups of people living in close proximity. Focusing on distinct household risk factors can help inform public health interventions that are able to prevent a comparatively larger number of cases.

Despite the unequal worldwide distribution of *H. pylori* infection, previous studies on risk factors for childhood infection have almost mostly been carried out in industrialized countries [10–15]. More research needs to be done to identify and compare risk factors in children in more resource-limited settings, as these are the demographics with the highest rates of infection. *H. pylori* prevalence is comparatively understudied in many African countries, though the studies that have been completed show higher than average prevalence rates [3, 4, 17, 18]. This makes studying childhood *H. pylori* infection in these countries important for disease surveillance and intervention. Understanding which contributing factors are inherent or mutable on the personal and household levels will enable public health workers to effectively maximize prevention by adding important clarifying details to the preexisting body of knowledge regarding *H. pylori* infections. This cross-sectional study therefore aimed to determine the magnitude of *H. pylori* infection among schoolchildren in Ziway, central Ethiopia and provide evidence for distinct personal and household risk factors associated with positive *H. pylori* childhood infection status.

## Methods

### Study area and design

This study was conducted in Ziway, Ethiopia along Lake Ziway located 160 km south of the country capital, Addis Ababa. Ziway has a population of 43,660 per the 2007 census and has experienced population and economic growth since its establishment in 1961. The population of Ziway is majority male (52.6%) and Orthodox Christian (51.0%). Participant study eligibility included children less than 15 years old who lived in Ziway and were enrolled in primary school. Data was collected from June 6 to July 30, 2016 at four different locations: Sher Elementary School, Sher Ethiopia Hospital, Batu Hospital, and Batu Health Clinic. A cross-sectional study was designed to examine the prevalence of *H. pylori* in Ziway schoolchildren as well as assess risk factors for acquiring *H. pylori* infection. A convenient sampling technique was used in order to enroll participants in the study; a child’s school attendance on a particular day determined participation. Enrollment in mass deworming programs is based on World Health Organization (WHO) recommendation, in which anthelmintic drugs are administrated to all schoolchildren regardless of their demographic difference.

### Data collection and laboratory analyses

Informed consent forms were signed by the parents of children involved in the study. Demographic and lifestyle information was collected through a questionnaire developed previously [19], and administered to the parents by a trained interviewer. Demographic questions included information on mother’s education, occupation, and place of residence. Lifestyle information included household size, sanitary conditions, child health practices, and latrine usage. Deworming status was ascertained from school records. Active *H. pylori* infection was determined using an antigen rapid test enzyme immunoassay (Human Helicobacter Pylori Rapid Test Cassette Antigen RapiCard InstaTest, Diagnostic Automation Inc., USA). Each participant was given a leak-proof plastic container and clear instructions on how to collect a fecal sample. Once collected, samples were placed in a container with 10% formalin and transported to Sher Ethiopia Hospital for detection of *H. pylori* antigens. For this, a small amount of stool was homogenized with buffer solution and added to test wells. A monoclonal mix of anti-*H. pylori* antibodies was used for the capture antibody and a mix of monoclonal peroxidase-conjugated anti-*H. pylori* antibodies was used for the detection antibody. The test was read after 15 min of contact with antibodies. A positive result was defined as the presence of two lines, even if the test line was noticeably fainter than the control line. A negative result was defined as the presence of only the control line. The sensitivity and specificity of the test are 94 and 96.7%, respectively.

An antibody rapid test (Diagnostic Automation Inc., USA) was used to detect any past or present *H. pylori* infections through a double antigen chromatographic lateral flow immunoassay. This test is unable to distinguish between current and past infection. A 5 mL whole blood sample was collected from each participant using sterile disposable syringes. Within two hours of blood collection, serum samples were separated at Sher Hospital laboratory to detect presence of *H. pylori* antibodies. For this assay, each test well received 1–2 drops of serum and was read 15 min after the serum was added. A positive result was defined as the presence of two lines, even if the test line was noticeably fainter than the control line. A negative result was defined as the presence of only the control line.

### Exposure definitions

Both personal and household-level risk factors were examined in order to understand the different levels of risk for *H. pylori* infection. Personal factors are individual attributes or behaviors that can affect the likelihood of developing a disease. Personal variables examined in this study include, but are not limited to, age, sex, medication history, and laboratory test results. All individual variables were able to be measured directly from participants.

Household factors are familial living conditions that affect health opportunities and outcomes for all people living in the same household. Some of the household factors assessed include, but are not limited to, indicators of socioeconomic status, housing conditions, cooking practices, water source, sanitation, and animal ownership. Several household factors are difficult to measure directly, so in these cases proxy measurements were used as estimates. Socioeconomic status was determined using maternal education and household electricity use. Quality of housing materials was defined by wall type and floor type. Housing conditions were evaluated by the presence of a bed for the child and household cooking location. Sanitation was assessed through type of latrine in household and materials used for cooking.

### Outcome definitions

A positive stool antigen rapid test indicates bacterial antigens in the body and is used in this study to define current *H. pylori* infection. A positive antibody rapid test from a blood serum sample indicates antibodies for the bacteria are present in the bloodstream following infection and is used in this study to define past infection. Positive *H. pylori* infection status is classified as a positive outcome for either test signifying *H. pylori* infection at some point during the child’s life.

### Statistical analysis

Data were entered, cleaned, and coded for analysis using SPSS Statistics (IBM Corp., Armonk, N.Y., USA). Descriptive statistics were used to characterize data and assess distribution of study variables. Univariate logistic regressions were completed in order to identify factors to include in further analysis. Multivariate logistic regressions were completed to assess the relationships between potential risk factors and *H. pylori* infection status after adjusting for confounding variables. Both direct multivariate analysis using all factors and stepwise regression including only variables associated with positive *H. pylori* infection status at *p* < 0.3 during univariate analysis were performed. Odds ratios were calculated to determine the magnitude of associations for each risk factor. Statistical significance was defined as *p* < 0.05. Similar patterns of demographic and lifestyle distributions were observed among study subject who had complete outcome data and all respondents using sensitivity analysis (data not shown).

### Data quality assurance

Questionnaires were inspected by two separate reviewers for completeness and consistency of biological data. Incomplete surveys were returned to participants to complete. Physical samples were analyzed immediately after collection. Two senior lab technicians read the data. Quality control samples were used for both the rapid antigen test and rapid antibody test.

## Results

### Sociodemographic distribution of study participants

A total of 434 schoolchildren participated in this study. The distribution of personal and household factors within the study population is summarized in Table 1. Of the children who participated, 7.90% were less than 5 years old, 48.7% ages 5–9, and 43.4% ages 10–14. A slight majority were male (50.5%) and most were not currently taking antibiotics (66.4%). Urban living was most common (96.3%) so the majority of families used electricity in the household (94.9%), although 73.7% of families cooked with natural materials and only 21.0% with gas or electricity. About 40% of households were classified as having poor sanitation, though 94.9% had a traditional pit or no toilet. Animal ownership was common (63.4%) with 42.6% of households owning a cat or dog and 34.6% owning a farm animal. Only a small percent (4.2%) of children lived in a house with a cigarette smoker.

**Table 1:** Distribution of sociodemographic factors in schoolchildren in Ziway, central Ethiopia

Personal Factors:	N	%
Age		
< 5	34	7.90
5–9	210	48.7
10–14	187	43.4
Sex		
Male	219	50.5
Female	215	49.5
Have older siblings		
Yes	265	61.2
No	168	38.8
Vaccination status		
Vaccinated	426	98.2
Unvaccinated	8	1.80
Regularly breastfeeding		
Yes	138	35.7
No	249	64.3
Currently taking antibiotics		
Yes	146	33.6
No	288	66.4
Deworming medication in last 6 months^a^		
Yes	313	72.1
No	121	27.9
Hemoglobin count g/dL		
< 11.5	17	3.90
≥ 11.5	417	96.1
**Household Factors:**		
Maternal education		
Formal	253	58.3
Non-formal	181	41.7
Maternal occupation		
Housewife	197	45.4
Employed	237	54.6
Socioeconomic status^b^		
Good	245	56.5
Poor	189	43.5
Lack of paracetamol^c^		
Yes	28	6.50
No	406	93.5
Residence location		
Rural	16	3.70
Urban	418	96.3
Number of people in house		
2–5	282	65.0
> 5	152	35.0
Smokers in house		
Yes	18	4.20
No	414	95.8
Housing materials^d^		
Good	369	85.0
Poor	65	15.0
Housing conditions^e^		
Good	384	88.5
Poor	50	11.5
Cooking location		
Inside house	332	76.7
Outside house	101	23.3
Cooking with natural materials^f^		
Yes	320	73.7
No	114	26.3
Gas or electric cooking		
Yes	91	21.0
No	343	79.0
Any electricity use in household		
Yes	412	94.9
No	22	5.10
Cat or dog ownership		
Yes	185	42.6
No	249	57.4
Farm animal ownership^g^		
Yes	150	34.6
No	284	65.4
Any animal ownership		
Yes	275	63.4
No	159	36.6
Main water source		
Piped	419	96.5
Well/natural water source	15	3.50
Type of toilet		
Flush or ventilated pit	22	5.10
Traditional pit or none	409	94.9
Waste disposal		
Pit/field/other	94	21.7
Burning/garbage bin	340	78.3
Sanitation^h^		
Good	260	59.9
Poor	174	40.1

^a^Deworming status was defined, received single dose (500 mg of mebendazole) as part of the mass school-based deworming programs in the past 6 months as evidenced form school records

^b^good socioeconomic status defined as formal maternal education and any household electricity use

^c^lack of paracetamol defined as lack of affordability or availability

^d^poor housing materials defined as mud and wood walls and uncovered mud floor

^e^poor housing conditions defined as no child bed and cooking inside main living area in house

^f^natural materials defined as dung, leaves, and wood

^g^farm animals defined as hen, cow/ox, sheep, horse, goat, pig, and mule/donkey

^h^poor sanitation defined as tradition pit or no toilet and using dung for cooking

### Prevalence of *H. pylori* infection

Positive *H. pylori* infection status was defined as either current or previous infection. As seen in Table 2, the prevalence of *H. pylori* infection among tested schoolchildren was 65.7% (285/434).

**Table 2:** Prevalence of *H. pylori* infection among schoolchildren in Ziway, central Ethiopia

***H. pylori*** status	N	%
Positive^a^	285	65.7
Negative	149	34.3

^a^positive infection status defined as positive result for either antibody or antigen test

### Association of Personal Factors and *H. pylori* infection

The crude associations between personal factors and *H. pylori* infection were assessed using univariate logistic regressions (Table 3). Of all the personal variables examined, univariate analysis found that the age group 10–14 showed statistically significant increased odds of infection (COR = 2.22, 95% CI: 1.06–4.66, *p* = 0.03), while age group 5–9 demonstrated a borderline significant correlation (COR = 1.88, 95% CI: 0.91–3.89, *p* = 0.09). Females (COR = 1.32, 95% CI: 0.89–1.97, *p* = 0.20) and those currently taking antibiotics (COR = 1.36, 95% CI: 0.90–2.07, *p* = 0.14) were also both associated with higher odds of *H. pylori* infection, though both failed to reach statistical significance.

**Table 3:** Associations between personal factors and *H. pylori* infection status in schoolchildren in Ziway, central Ethiopia

	Positive	***H. pylori***	Negative	***H. pylori***									
	N	%	N	%	COR	(95% CI)	P	AOR	(95% CI)	P	AOR^**a**^	(95% CI)	P
Age													
< 5	17	50.0	17	50.0	1	–	–	1	–	–	1	–	–
5–9	137	65.2	73	34.8	1.88	(0.91–3.89)	0.09	1.77	(0.81–3.87)	0.15	1.82	(0.87–3.80)	0.11
10–14	129	69.0	58	31.0	2.22	(1.06–4.66)	**0.03**	1.98	(0.88–4.44)	0.10	2.07	(0.98–4.39)	0.06
Sex													
Male	137	62.6	82	37.4	1	–	–	1	–	–	1	–	–
Female	148	68.8	67	31.2	1.32	(0.89–1.97)	0.20	1.36	(0.90–2.04)	0.15	1.34	(0.89–2.00)	0.16
Have older siblings													
Yes	169	63.8	96	36.2	0.81	(0.54–1.22)	0.32	0.76	(0.50–1.16)	0.21	–	–	–
No	115	68.5	53	31.5	1	–	–	1	–	–	–	–	–
Vaccination status													
Vaccinated	280	65.7	146	34.3	1	–	–	1	–	–	–	–	–
Unvaccinated	5	62.5	3	37.5	0.87	(0.20–3.69)	0.85	2.13	(0.38–12.1)	0.39	–	–	–
Regularly breastfeeding													
Yes	95	36.4	43	34.1	1	–	–	1	–	–	–	–	–
No	166	63.6	83	65.9	0.91	(0.58–1.42)	0.66	0.93	(0.59–1.46)	0.74	–	–	–
Currently taking antibiotics													
Yes	89	61.0	57	39.0	1	–	–	1	–	–	1	–	–
No	196	68.1	92	31.9	1.36	(0.90–2.07)	0.14	1.24	(0.80–1.91)	0.34	1.26	(0.82–1.93)	0.29
Deworming medication in last 6 months													
Yes	204	65.2	109	34.8	0.92	(0.59–1.44)	0.73	0.91	(0.57–1.44)	0.68	–	–	–
No	81	66.9	40	33.1	1	–	–	1	–	–	–	–	–
Hemoglobin count g/dL													
< 11.5	10	58.8	7	41.2	0.74	(0.28–1.98)	0.55	0.70	(0.22–2.25)	0.55	–	–	–
≥ 11.5	275	65.9	142	34.1	1	–	–	1	–	–	–	–	–

^a^AOR includes only variables with COR p < 0.3

Direct and stepwise multivariate logistic regressions were performed in order to determine any associations with *H. pylori* infection after adjusting for confounding factors. Direct multivariate analysis yielded similar trends as in univariate analysis, except none of the associations in this regression model reached statistical significance (Table 3). Vaccination status was the only variable with an OR that greatly changed from crude to adjusted, with unvaccinated children showing a moderately strong non-significant association with higher odds of *H. pylori* infection (AOR = 2.13, 95% CI: 0.38–12.1, *p* = 0.39). Stepwise multivariate analysis including only individual factors with a COR *p* < 0.3 also yielded no statistically significant results, but again demonstrated that older age, female sex, and current antibiotic use were correlated with higher odds of *H. pylori* infection. Most ORs remained relatively stable throughout univariate and multivariate analysis.

### Association of Household Factors and *H. pylori* infection

The crude associations between household-level factors and *H. pylori* infection were assessed using univariate logistic regressions. Out of all household variables assessed, univariate analysis found that having a traditional pit or no toilet was the only factor associated with statistically significant increased odds of *H. pylori* infection (COR = 3.59, 95% CI: 1.47–8.77, *p* = 0.01). Poor sanitation (COR = 1.46, 95% CI: 0.95–2.25, *p* = 0.08) and no household electricity use (COR = 1.87, 95% CI: 0.66–5.05, *p* = 0.25) were also correlated with slightly higher odds of infection, though failed to reach statistical significance. Conversely, cigarette smokers living in the household significantly decreased the odds of positive *H. pylori* infection status (COR = 0.32, 95% CI: 0.12–0.84, *p* = 0.02). Rural dwelling (COR = 0.51, 95% CI: 0.19–1.39, *p* = 0.19) and cooking with natural materials (COR = 0.72, 95% CI: 0.45–1.14, *p* = 0.16) were both associated with statistically insignificant decreased odds of *H. pylori* infection.

Direct and stepwise multivariate logistic regressions were performed in order to determine associations with *H. pylori* infection after adjusting for confounding factors (Table 4). Direct multivariate analysis yielded similar trends as in univariate analysis, with traditional pit or no toilet being significantly associated with positive *H. pylori* status (AOR = 3.78, 95% CI: 1.40–10.3, *p* = 0.01) and presence of household smokers significantly associated with negative infection status (AOR = 0.35, 95% CI: 0.12–0.99, *p* = 0.05). Poor socioeconomic status (AOR = 3.85, 95% CI: 0.29–50.8. *p* = 0.31), cat or dog ownership (AOR = 2.15, 95% CI: 0.98–4.72, *p* = 0.06), and farm animal ownership (AOR = 2.03, 95% CI: 0.72–5.70, *p* = 0.18) all showed increased moderate strengths of association with positive *H. pylori* infection status after adjustment, though failed to reach statistical significance. Informal maternal education (AOR = 0.39, 95% CI: 0.03–5.05, *p* = 0.47), rural residence (AOR = 0.40, 95% CI: 0.12–1.33, *p* = 0.14), and any animal ownership (AOR = 0.45, 95% CI: 0.17–1.15, *p* = 0.09) all showed statistically insignificant decreased odds of infection. Household factors with a COR *p* < 0.3 were further examined in stepwise multivariate analysis to find similar results. Having a traditional pit or no toilet was correlated with 3.93-fold higher odds of *H. pylori* infection (AOR = 3.93, 95% CI: 1.51–10.3, *p* = 0.01), while the presence of cigarette smokers in the household demonstrated 68% lower odds of infection (AOR = 0.32, 95% CI: 0.11–0.89, *p* = 0.03). Disposing of waste in pits or fields (COR = 0.61, 95% CI: 0.37–1.02. *p* = 0.06) showed a borderline significant decrease in odds of infection. Associations were generally strengthened from univariate analysis to multivariate adjustment.

**Table 4:** Associations between household factors and *H. pylori* infection status in school children in Ziway, central Ethiopia

	Positive	***H. pylori***	Negative	***H. pylori***									
	N	%	N	%	COR	(95% CI)	P	AOR	(95% CI)	P	AOR^**a**^	(95% CI)	P
Maternal education													
Formal	162	64.0	91	36.0	1	–	–	1	–	–	–	–	–
Non-formal	123	68.0	58	32.0	1.19	(0.80–1.79)	0.40	0.39	(0.03–5.05)	0.47	–	–	–
Maternal occupation													
Housewife	136	69.0	61	31.0	1.32	(0.88–1.97)	0.18	1.24	(0.81–1.91)	0.32	1.27	(0.84–1.94)	0.26
Employed	149	62.9	88	37.1	1	–	–	1	–	–	1	–	–
Socioeconomic status^b^													
Good	155	63.3	90	36.7	1	–	–	1	–	–	1	–	–
Poor	130	68.8	59	31.2	1.29	(0.86–1.91)	0.23	3.85	(0.29–50.8)	0.31	1.33	(0.86–2.06)	0.21
Lack of paracetamol^c^													
Yes	19	67.9	9	32.1	1.11	(0.49–2.52)	0.80	1.05	(0.42–2.59)	0.92	–	–	–
No	266	65.5	140	34.5	1	–	–	1	–	–	–	–	–
Residence location													
Rural	8	50.0	8	50.0	0.51	(0.19–1.39)	0.19	0.40	(0.12–1.33)	0.14	0.39	(0.12–1.25)	0.11
Urban	277	66.3	141	33.7	1	–	–	1	–	–	1	–	–
Number of people in house													
2–5	182	64.5	100	35.5	1	–	–	1	–	–	–	–	–
> 5	103	67.8	49	32.2	1.16	(0.76–1.76)	0.50	1.19	(0.75–1.90)	0.45	–	–	–
Smokers in house													
Yes	7	38.9	11	61.1	0.32	(0.12–0.84)	**0.02**	0.35	(0.12–0.99)	**0.05**	0.32	(0.11–0.89)	**0.03**
No	276	66.7	138	33.3	1	–	–	1	–	–	1	–	–
Housing materials^d^													
Good	240	65.0	129	35.0	1	–	–	1	–	–	–	–	–
Poor	45	69.2	20	30.8	1.21	(0.69–2.14)	0.51	1.37	(0.73–2.59)	0.33	–	–	–
Housing conditions^e^													
Good	251	65.4	133	34.6	1	–	–	1	–	–			
Poor	34	68	16	32	1.13	(0.60–2.12)	0.71	0.98	(0.49–1.94)	0.95	–	–	–
Cooking location											–	–	–
Inside house	222	66.9	110	33.1	1	–	–	1	–	–			
Outside house	62	61.4	39	38.6	0.79	(0.50–1.25)	0.31	0.69	(0.40–1.19)	0.18	–	–	–
Cooking with natural materials^f^											–	–	–
Yes	204	63.7	116	36.3	0.72	(0.45–1.14)	0.16	0.65	(0.34–1.24)	0.19	0.65	(0.39–1.09)	0.10
No	81	71.1	33	28.9	1	–	–	1	–	–	1	–	–
Gas or electric cooking													
Yes	62	68.1	29	31.9	1	–	–	1	–	–	–	–	–
No	223	65.0	120	35.0	0.87	(0.53–1.42)	0.58	0.90	(0.45–1.80)	0.77	–	–	–
Any electricity use in household													
Yes	268	65.0	114	35.0	1	–	–	1	–	–	1	–	–
No	17	77.3	5	22.7	1.87	(0.66–5.05)	0.25	1.94	(0.47–7.98)	0.36	2.53	(0.76–8.51)	0.13
Cat or dog ownership													
Yes	125	67.6	60	32.4	1.16	(0.78–1.73)	0.47	2.15	(0.98–4.72)	0.06	–	–	–
No	160	64.3	89	35.7	1	–	–	1	–	–	–	–	–
Farm animal ownership^g^													
Yes	105	70.0	45	30.0	1.35	(0.88–2.06)	0.17	2.03	(0.72–5.70)	0.18	1.16	(0.51–2.68)	0.72
No	180	63.4	104	36.6	1	–	–	1	–	–	1	–	–
Any animal ownership													
Yes	183	66.5	92	33.5	1.11	(0.74–1.67)	0.61	0.45	(0.17–1.15)	0.09	–	–	–
No	102	64.2	57	35.8	1	–	–	1	–	–	–	–	–
Main water source													
Piped	276	65.9	143	34.1	1	–	–	1	–	–	–	–	–
Well/natural water source	9	60.0	6	40.0	0.78	(0.27–2.23)	0.64	0.65	(0.17–2.59)	0.55	–	–	–
Type of toilet													
Flush or ventilated pit	8	36.4	14	63.6	1	–	–	1	–	–	1	–	–
Traditional pit or none	275	67.2	134	32.8	3.59	(1.47–8.77)	**0.01**	3.78	(1.40–10.3)	**0.01**	3.93	(1.51–10.3)	**0.01**
Waste disposal													
pit/field/other	54	57.4	40	42.6	0.64	(0.40–1.02)	0.06	0.61	(0.36–1.04)	0.07	0.61	(0.37–1.02)	0.06
burning/garbage bin	231	67.9	109	32.1	1	–	–	1	–	–	1	–	–
Sanitation^h^													
Good	181	62.8	107	37.2	1	–	–	1	–	–	1	–	–
Poor	104	71.2	42	28.8	1.46	(0.95–2.25)	0.08	1.10	(0.46–2.60)	0.83	1.15	(0.50–2.62)	0.75

^a^AOR includes only variables with COR p < 0.3

^b^good socioeconomic status defined as formal maternal education and any household electricity use

^C^ack of paracetamol defined as lack of affordability or availability

^d^poor housing materials defined as mud and wood walls and uncovered mud floor

^e^poor housing conditions defined as no child bed and cooking inside main living area in house

^f^natural materials defined as dung, leaves, and wood

^g^farm animals defined as hen, cow/ox, sheep, horse, goat, pig, and mule/donkey

^h^poor sanitation defined as tradition pit or no toilet and using dung for cooking

## Discussion

This study identified the prevalence of *H. pylori* infection in central Ethiopian schoolchildren and provides additional evidence for associations between distinct personal and household correlates and infection status. Positive infection status was significantly associated with older age at the individual level and having a traditional pit or no toilet at the household level. Presence of smokers in the household showed statistically significant decreased odds of infection. There was a borderline significant negative correlation between infection and pit or field waste disposal as well.

The *H. pylori* prevalence rate in schoolchildren in Ziway, Ethiopia was 65.7%, signifying that over half of children less than 15 years old have been infected at some point in their life. This rate is higher than reports of the average global prevalence [3, 4], though comparable to recent studies in rural Ethiopia [17, 18]. However, previously reported rates are inclusive of all age ranges, not just school-aged children. Assuming the trend of increased infection with older age continues, this suggests an overall higher disease prevalence in central Ethiopia than calculated in this study.

Of all the personal variables assessed, older age was associated with higher odds of positive *H. pylori* infection status, with ages 10–14 having a stronger association than ages 5–9. Older age is a common risk factor for *H. pylori* infection cited in previous studies [9, 13–15, 18, 20–24]. There is no consensus yet whether this trend is due to cumulative increased risk over a lifetime [11, 22] or decreased risk of infection in each subsequent birth cohort causing older cohorts to have higher infection rates comparatively [20, 24]. Due to the nature of this study, the data presented here cannot support one hypothesis over the other.

Of all the household-level factors investigated, having a traditional pit or no toilet at all was significantly associated with 3.93-fold higher odds of *H. pylori* infection after adjusting for confounding factors. To date, contradictory literature exists regarding associations between latrine use and *H. pylori* infection status [14, 18]. Having no toilet or a traditional pit could increase the possibility of contact with fecal material as compared to flush toilets or ventilated pits. In a study on traditional pit sanitation in Malawi, it was found that traditional pits can promote transmission of fecal-oral diseases due to lack of maintenance, poor disposal of feces, fouled wash-water, and potential for pit surcharging [25]. Flush toilets, on the other hand, have been proposed as a mechanism for disease vector removal, which can help prevent further infection [14]. Since *H. pylori* can be detected in fecal material [7, 8], using traditional pits or no toilet may increase the potential for contact with the bacteria and facilitate infection. The significant association between toilet type and infection status reported in this study thus supports the proposed fecal-oral route of transmission for *H. pylori*. Accordingly, hygiene education and availability of alternative toilet types are potential areas of focus for disease control and prevention.

Strikingly, at the household level the presence of cigarette smokers in the household was significantly correlated with 68% decreased odds of *H. pylori* infection after adjusting for confounding factors. Previous studies have published conflicting results on the relationship between smoking as a personal attribute and *H. pylori* infection status [18, 26], though there is little data addressing infection rates in those living with smokers. In other developing African countries, smoking is more common among wealthy people [27]. Considering the small number of children in this study with family members that smoke, the presence of smokers in the household could thus be indicative of good socioeconomic status. Poorer socioeconomic status has been previously reported to be associated with increased *H. pylori* infection [11, 13, 21, 23], perhaps due to limited access to food, water, healthcare, and soap, among other things. This suggests that poverty and access to education may be possible targets for *H. pylori* prevention. Yet, as another explanation, Ogihara et al. proposed that chemical interactions between nicotine smoke and gastric mucosa can increase gastric acidity and mucosal atrophy, assisting in the auto-eradication of *H. pylori* infections [26]. It is unknown whether secondhand smoke exposure can lead to similar results. It is also possible that if adults who smoke have lower prevalence rates, there is less potential for household transmission. However, since this study does not address cigarette smoking as a personal variable, it remains unclear how the presence of smokers in the household influences *H. pylori* infection status. The effects of smoking on bacterial transmission in parents and children warrants further investigation.

Additionally, pit or field waste disposal was borderline significantly associated with decreased odds of positive *H. pylori* infection status as compared to burning or using garbage bins. A previous study in Asella, central Ethiopia describes limited access to door-to-door waste collection services in Ethiopian towns outside of the capital city [28]. Waste disposed in garbage bins in Ziway may thus not be frequently collected and instead stored in the home for extended periods of time. A study in Ghana found that storing solid waste in open containers in the home increased the presence of houseflies, which was in turn associated with increased gastrointestinal disease occurrence in children [29]. Similarly, Parente et al. found that the absence of garbage collection services is positively associated with *H. pylori* infection [30]. Houseflies are known vectors for disease transmission from fecal matter to food. It is thus possible that garbage bin waste disposal increases vector-borne transmission of *H. pylori*, again aligning with the proposed fecal-oral route of transmission. However, further investigation on waste management and disposal practices is needed to explore this possibility.

These findings should be interpreted in light of a few study limitations. First, a cross-sectional study design was used as there were no previously available baseline data on *H. pylori* infection status. Because of the observational nature of this study, it is difficult to conclude temporality and causality between personal and household variables and infection. A longitudinal study is needed to confirm our findings and assess the possibility of reverse causality. Additionally, only a small region of central Ethiopia was sampled, limiting the generalizability of the reported prevalence rates to the entire country. It is also probable that some residual confounding personal or household factors were excluded from analysis despite the large number of variables examined. Surveying the parents instead of the participants themselves limited the collection of information on child-specific variables such as frequency of handwashing after using the latrine or whether a child helps with food preparation. Our study participants were apparently healthy children enrolled in primary school, so the observed individual and household risk factors for *H pylori* infection might be different if we included children who dropped out or never attended the school. A further limitation of the current study is that some subgroup categories had small sample sizes which led to wide and imprecise confidence intervals for those observed measures of association.

Despite these limitations, this study is the first to differentiate between personal- and household-level risk factors for *H. pylori* infection in a developing country. This distinction allows for a more thorough assessment of disease transmission and determination of which prevention efforts are most useful for disease control. The high rate of participation and large sample size used serve to strengthen the study findings. Additionally, because separate antigen and antibody rapid tests were performed, analyses were able to assess risk factors for both current and past infection.

## Conclusion

Our data from a developing country provides additional support for older age as a personal risk factor for *H. pylori* infection and identifies correlations between socioeconomic and sanitation household factors and positive childhood infection status. The associations reported here support the hypothesized fecal-oral route of transmission for *H. pylori.* Further longitudinal research is needed to establish causality and exact mechanisms.

## Data Availability

The datasets analyzed in the current study will be available from the corresponding author upon reasonable request.

## Abbreviations

*H. pylori*: Helicobacter pylori
WHO: World Health Organization
